# Error Propagation in Microwave Soil Moisture and Vegetation Optical Depth Retrievals

**DOI:** 10.1109/jstars.2021.3124857

**Published:** 2021-11-13

**Authors:** Andrew F. Feldman, David Chaparro, Dara Entekhabi

**Affiliations:** Department of Civil and Environmental Engineering, Massachusetts Institute of Technology, Cambridge, MA 02139 USA.; Department of Civil and Environmental Engineering, Massachusetts Institute of Technology, Cambridge, MA 02139 USA.; CommSensLab, Institut d’Estudis Espacials de Catalunya, Universitat Politècnica de Catalunya, 08034 Barcelona, Spain.

**Keywords:** Brightness temperature, microwave retrieval algorithms, regularization, soil moisture, vegetation optical depth

## Abstract

Satellite soil moisture and vegetation optical depth [(VOD); related to the total vegetation water mass per unit area] are increasingly being used to study water relations in the soil-plant continuum across the globe. However, soil moisture and VOD are typically jointly estimated, where errors in the optimization approach can cause compensation between both variables and confound such studies. It is thus critical to quantify how satellite microwave measurement errors propagate into soil moisture and VOD. Such a study is especially important for VOD given limited investigations of whether VOD reflects *in situ* plant physiology. Furthermore, despite new approaches that constrain (or regularize) VOD dynamics to reduce soil moisture errors, there is limited study of whether regularization reduces VOD errors without obscuring true vegetation temporal dynamics. Here, we find that, across the globe, VOD is less robust to measurement error (more difficult for optimization methods to find the true solution) than soil moisture in their joint estimation. However, a moderate degree of regularization (via time-constrained VOD) reduces errors in VOD to a greater degree than soil moisture and reduces spurious soil moisture-VOD coupling. Furthermore, despite constraining VOD time dynamics, regularized VOD variations on subweekly scales are both closer to simulated true VOD time series and have global VOD post-rainfall responses with reduced error signatures compared to VOD retrievals without regularization. Ultimately, we recommend moderately regularized VOD for use in large scale studies of soil-plant water relations because it suppresses noise and spurious soil moisture-VOD coupling without removing the physical signal.

## I. Introduction

Vegetation optical depth (VOD) and soil moisture, as remotely measured from microwave spaceborne sensors, are now widely used across geophysical investigations. These quantities are often jointly estimated using satellite measurements, for example, from low frequency microwave (L-band; 1.4 GHz) sensors onboard the Soil Moisture and Ocean Salinity (SMOS) and Soil Moisture Active Passive (SMAP) satellites [1], [2]. VOD is directly related to the total water mass within the vegetation canopy per unit area (traditionally named vegetation water content) [3]. As a quantity that is challenging to measure *in situ* across large spatial scales, satellite VOD can be used to understand vegetation’s role in the global water and carbon cycles via vegetation phenological cycles, plant hydraulics, and terrestrial carbon storages [4], [5]. Soil moisture is a small, but highly active global water reservoir that links the water, carbon, and energy cycles at the land-atmosphere interface [6]. The joint study of these variables across lower microwave frequencies is enabling an improved understanding of soil-plant water relations and fluxes through the soil-plant-atmosphere continuum [4], [7]–[10]. These studies ultimately rely on how well both soil moisture and VOD can be jointly retrieved from satellite instruments. Despite widespread use of both variables, only uncertainty of soil moisture alone has largely been assessed while VOD and joint soil moisture-VOD uncertainty assessments are largely absent from the literature.

An accurate assessment of errors in both variables is essential to investigate temporal dynamics of soil and plants individually and jointly. While VOD is increasingly used in ecosystem studies [4], [5], [11]–[13], it is unknown how well satellite VOD reflects *in situ* plant temporal dynamics. Though annual average VOD was found to correspond well with aboveground biomass [5], [14], VOD *in situ* investigations of daily to seasonal dynamics are only sparsely becoming available due to the difficulty of obtaining time-dynamic field vegetation measurements related to VOD at large spatial scales [15]–[19]. Other satellite vegetation products, such as leaf area index or solar induced fluorescence, are limited in validating VOD because they measure vegetation at optical and thermal frequencies and detect canopy features only partially related to VOD [4], [20]. With *in situ* VOD measurements available only at sparse field sites, broader generalizations of VOD error quantification are currently limited to evaluation of algorithmic VOD uncertainty. Moreover, satellite-based soil moisture dynamics broadly show consistency with soil measurements at more expansive calibration/validation field sites [21], [22]. However, misrepresenting VOD dynamics has been shown to drive soil moisture errors, which conveys the detriment of evaluating soil moisture uncertainty in isolation of VOD considerations [23]–[27]. This is because microwave emission used to retrieve both quantities is a strong function of both of these variables (via the surface roughness and canopy transmissivity at microwave frequencies) [26], [28], [29]. As such, it is critical to quantify the degree to which satellite measurement error propagates simultaneously into soil moisture and VOD retrievals within retrieval algorithms and identify where artifacts arise.

Soil moisture and VOD are traditionally retrieved simultaneously where both variables are estimated from horizontally and vertically polarized microwave brightness temperature measurements (TB_H_ and TB_V_, respectively) at each satellite overpass. Such retrieval algorithms, referred to here as simultaneous retrieval approaches, include the dual channel algorithm (DCA) and land parameter retrieval model (LPRM) [22], [30], [31]. However, it has been shown that raw satellite measurements of horizontally and vertically polarized TB are correlated and, thus, do not provide two full degrees of information [32]. This mutual information results in an inability to fully retrieve two unknowns regardless of the electromagnetic model used. As a result, optimization search instabilities occur, which cause errors when simultaneously estimating soil moisture and VOD [33], [34]. Furthermore, retrieved VOD using these simultaneous retrieval approaches has been noted as unreliable at short timescales [26], [35].

It is becoming common practice to reduce soil moisture and VOD errors in a procedure termed “regularization” [22], [34], [36], [37]. In this context, regularization solves underdetermined problems (i.e., using two dependent TB measurements to retrieve two unknowns) by imposing *a priori* information about soil moisture and/or VOD to enable a robust inversion. There are now increasingly-used algorithms that regularize VOD such as the multitemporal dual channel algorithm (MT-DCA), Sobolev-Norm, and Tikhonov regularization approaches [33], [34], [37], [38]. The MT-DCA regularizes on a discrete basis by retrieving VOD on a given overpass using TB information from time-adjacent overpasses [37]. Like the MT-DCA, the Sobolev-norm approach constrains VOD rates of change on a continuous-basis using a penalty term in the cost function. The Tikhonov approach constrains VOD variations to be similar to those of an input *a priori* VOD time series [33], [39]. These regularization approaches are now being implemented within widely-used satellite mission retrieval algorithms [36], [40], [41].

We focus here on regularization through constraining time derivatives of VOD such as using the MT-DCA and Sobolev-norm because they do not require *a priori* VOD time series to implement. Regularization in this manner presents a tradeoff. It adds *a priori* information on time dynamics of VOD that it is slower in time than soil moisture. This stabilizes the soil moisture-VOD parameter optimization search which reduces retrieval errors. However, constraining VOD changes to a large degree may partially remove real VOD temporal dynamics (especially at shorter timescales) beyond only errors [33]. Thus, a greater degree of regularization may result in greater retrieval error reduction, but potentially at the expense of subduing real VOD changes at shorter timescales. The goal is to moderately regularize VOD such that primarily VOD changes due to error are removed rather than true dynamics. We expect the optimal amount of regularization to improve and allow interpretation of VOD dynamics at subweekly timescales.

Smoothing a retrieved time series with a low-pass filter is not equivalent to the time derivative regularization discussed here. This is because smoothing explicitly removes short timescale variability without the benefits of stabilizing the optimization and suppressing errors by imposing *a priori* information [33].

The regularization approach discussed here that constrains the VOD time derivative assumes that the vegetation temporal dynamics that VOD represents occur slower than surface soil moisture changes. Consider that VOD has been shown to be a joint function of plant moisture and biomass changes across timescales [16], [42]. The assumption of slower VOD than soil moisture dynamics is viable because while plant saturation changes simultaneously with soil moisture under predawn water potential equilibrium [43], VOD contributions from plant dry biomass changes are slower than moisture changes and will, thus, act to slow the overall VOD signal relative to soil moisture [44].

Ultimately, it is unclear how errors propagate into soil moisture and VOD within these simultaneous and regularization retrieval approaches. It is similarly unknown how much regularization suppresses VOD errors and whether it allows better interpretation of the true VOD time dynamics. In this article, we ask: how does satellite measurement error propagate into soil moisture and vegetation optical depth? Can regularization reduce VOD noise without obscuring real VOD temporal dynamics? This article expands on recent work that evaluated retrieval noise reductions between simultaneous retrievals and regularization approaches [24], [33], [38]. These studies mainly focused on error reductions in soil moisture, whereas the work here provides increased focus on the VOD signal, its errors, and its interactions with soil moisture.

We refer to VOD as a radiative transfer parameter throughout and focus on the errors in its estimation in forward modeling the radiative transfer equations. VOD’s relation to any geophysical parameter and the functional forms of that relationship are topics for ecology and hydrology application studies. We note that VOD is known to be monotonically related to the total water mass per unit area and be sensitive to biomass and percent saturation, but its exact relation to canopy properties is still an open research question [3], [45].

The rest of this article is organized as follows. We first motivate the article by theoretically estimating error propagation into soil moisture and VOD in simultaneous retrieval frameworks in Section II. In Section III, we investigate the effects of VOD regularization on soil moisture and VOD errors. Finally, in Section IV, we evaluate the effects of soil moisture and VOD errors as well as regularization on the VOD signal.

## II. Error Quantification in Simultaneous Retrievals

We first investigate theoretically how satellite instrument brightness temperature measurement errors propagate into soil moisture and VOD in traditional simultaneous retrieval algorithms that do not use regularization. Previous global microwave parameter sensitivity analyses have been conducted but mainly focus on effects of soil moisture in numerical frameworks [28], [46], [47]. Though they tend to agree on high level of TB sensitivity to soil moisture over other parameters, it is unclear how this finding translates into error propagation, respectively, into these variables, especially VOD. The following evaluation only reflects the mathematical formulation of the zeroth-order radiative transfer model, or the so-called tau–omega model [48], not natural processes on the land surface. Using a different radiative transfer model would produce different results [49]. However, we expect these results to be widely relevant because the tau–omega model framework is applied across microwave land surface retrieval algorithms. The analysis here ultimately motivates Sections III and IV.

Soil moisture and VOD are most commonly estimated from TB_H_ and TB_V_ within the tau–omega model [48]. This can be accomplished using the DCA or LPRM. At this stage, TB measurement errors can propagate into soil moisture and VOD retrievals. One can estimate these errors and their properties using the Hessian matrix of the cost function. The typical cost function (J) for soil moisture and VOD retrieval is

(1) $$J=\frac{1}{2}[{\left(\frac{{\text{TB}}_{H\;\text{Obs}}-{\text{TB}}_{H\;\text{Mod}}}{\sigma_{{\text{TB}}_{H}}}\right)}^{2}+{\left(\frac{{\text{TB}}_{V\;\text{Obs}}-{\text{TB}}_{V\;\text{Mod}}}{\sigma_{{\text{TB}}_{V}}}\right)}^{2}]$$

where TB_Mod_ is forward modeled using the tau–omega model with the goal of reducing the squared differences with the observed TB (TB_Obs_) by adjusting mainly soil moisture and VOD. *σ* is the TB measurement error (*σ*_TBH_ and *σ*_TBV_) and is known to be normally distributed. We chose an error standard deviation of 1.1K in both polarizations independently, which is a conservative choice compared to NEDT estimations of approximately 0.9K–0.96K [50]. Note that the choice of NEDT does not qualitatively change results because it mainly applies a mean scaling to the errors but does not change the plotted relationships and conclusions.

**Fig. 1.**
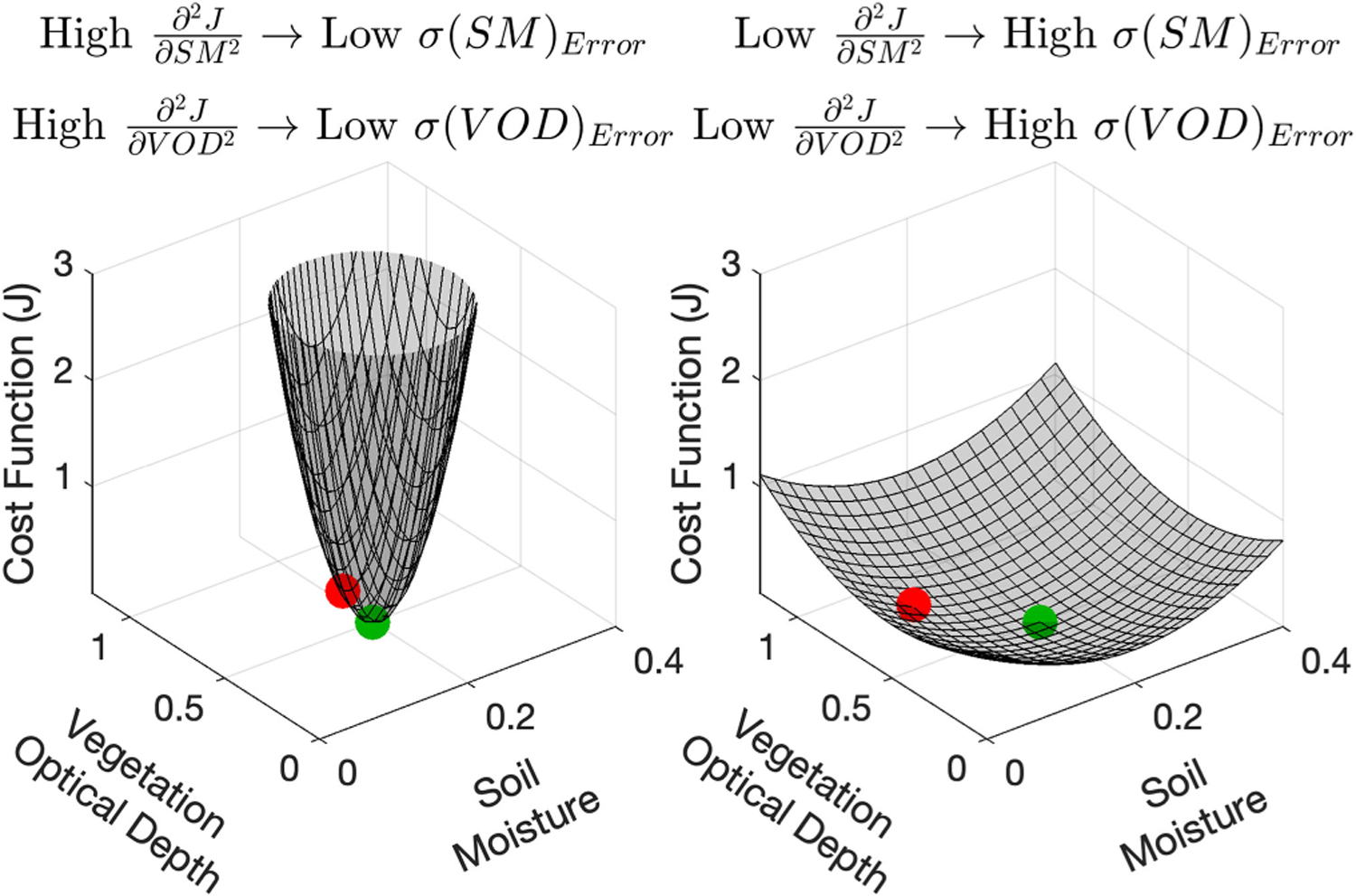
Schematic of cost function scenarios with resulting second derivatives and target variable variances (SM represents soil moisture; VOD represents vegetation optical depth). The true solution is shown by the green symbol and noisy retrieval by the red symbol. The deviation of the retrieval from the true solution represents the error. Estimation on the left panel is more robust to error (lower error standard deviation expressed as *σ*) than on the right panel because the second derivative magnitudes of the cost function are larger. The units of J are arbitrary here.

The second derivative of J with respect to soil moisture and VOD can isolate how robust soil moisture and VOD, respectively, are to errors (see Fig. 1). We use “robust” in this context to refer to how well the optimization approach can find the true solution as determined by the curvature of the cost function. Based on the curvature of the cost function, the inverse of the Hessian matrix (H) provides an estimate of the error variances of soil moisture and VOD when the variables are normally distributed and not strongly nonlinearly related [51]

(2) $$\Sigma =H^{-1}={\left[\begin{array}{cc} \frac{\partial^{2}J}{\partial SM^{2}} & \frac{\partial^{2}J}{\partial \text{SM}\partial \text{VOD}}\\ \frac{\partial^{2}J}{\partial \text{VOD}\partial \text{SM}} & \frac{\partial^{2}J}{\partial {\text{VOD}}^{2}} \end{array}\right]}^{-1}\;=\left[\begin{array}{cc} \sum_{\text{SM},\text{SM}} & \sum_{\text{SM},\text{VOD}}\\ \sum_{\text{VOD},\text{SM}} & \sum_{\text{VOD},\text{VOD}} \end{array}\right]$$

(3) $$\sigma {\left(\text{SM}\right)}_{\text{Error}}={\left(\Sigma_{\text{SM},\text{SM}}\right)}^{\frac{1}{2}}$$

(4) $$\sigma {\left(\text{VOD}\right)}_{\text{Error}}={\left(\Sigma_{\text{VOD,VOD}}\right)}^{\frac{1}{2}}$$

where Σ is the covariance matrix and the diagonals of Σ are the soil moisture and VOD error variance. The soil moisture and VOD error standard deviation, *σ*(SM)_Error_ and *σ*(VOD)_Error_, respectively, can, thus, be computed in (3) and (4). Although they model a more idealized error pattern and normality may not always hold, (3) and (4) provide a viable approximation that we use to evaluate error patterns within the simultaneous soil moisture and VOD estimation space.

As predicted from (2), Fig. 1 demonstrates that large second derivatives of the cost function equate to low variances of the estimated metrics. Specifically, the deep instead of shallow well allows optimization search methods to estimate the true solution more closely in the presence of noise. Conversely, a shallow well would result in gradient search methods having difficulty converging to the true solution. This creates undesirable variability within each variable as well as compensation between the variables from noise-based artifacts. Ultimately, joint variability of soil moisture and VOD that is due to error would bias true soil moisture-VOD relationships and lead to misinterpretations of their physical behavior.

**Fig. 2.**
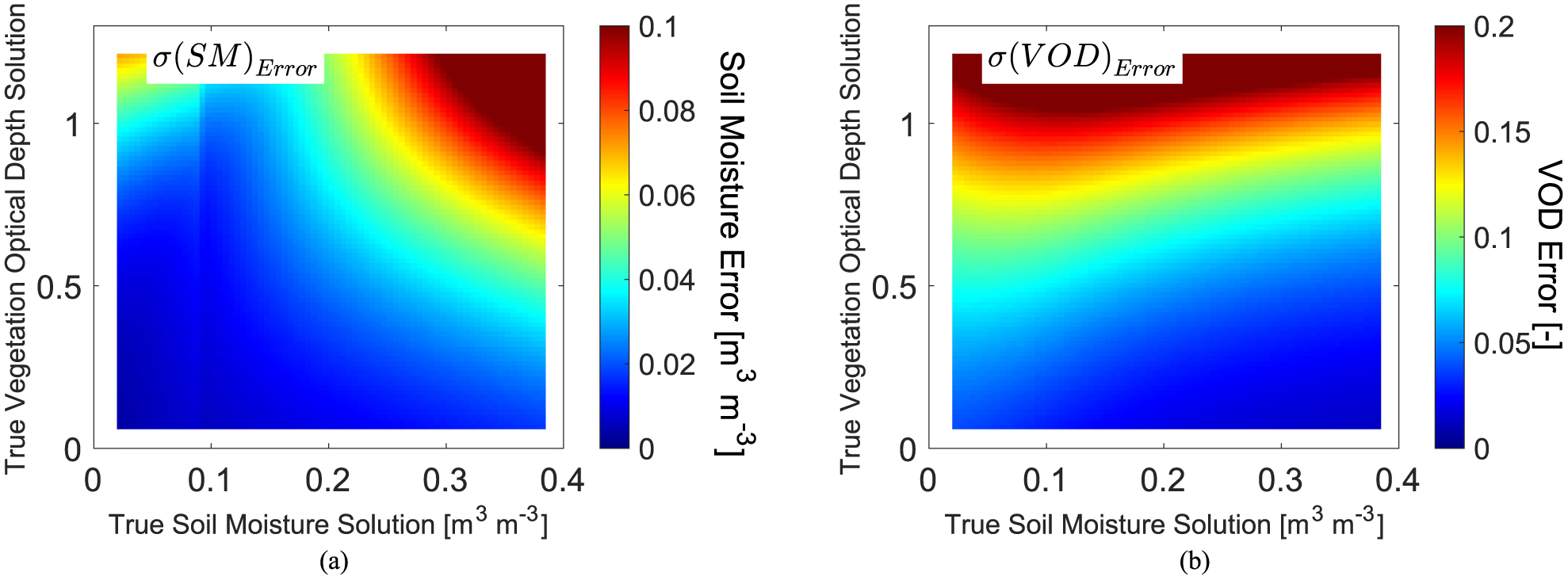
Inverse Hessian matrix estimated (A) soil moisture and (B) vegetation optical depth error standard deviations. Given a 1.1K TB error standard deviation in both polarizations, error standard deviation of soil moisture and VOD are shown at the true solution for each given soil moisture-VOD pair. For reference, grasslands and savannas have mean VOD values of 0.2–0.4 while forests have values above 0.6. SMAP’s mission goal is for a soil moisture unbiased root mean square difference [metric similar to that plotted in (A)] of less than 0.04 m^3^ m^−3^. SMAP flags soil moisture retrievals for poor quality above VOD of approximately 0.5 due to increasingly dense vegetation cover. The abrupt error change along the *x*-axis in (A) near 0.09 m^3^ m^−3^ originates from the Mironov soil mixing dielectric model in [52].

Next, the robustness of soil moisture and VOD across all true combinations of soil moisture and VOD are evaluated by numerically computing the soil moisture and VOD error standard deviation using the inverse Hessian matrix in (3) and (4) (see Fig. 2). Inverse Hessian matrices are numerically computed at each true soil moisture-VOD pair. Nominal values were chosen to model TB via the tau–omega model including a single scattering albedo of 0.1, a physical temperature of 290K, a clay fraction of 0.2, a surface roughness model as in [22], and the Mironov soil mixing dielectric model [52]. These same parameters are used throughout the study, unless otherwise specified. While the magnitudes can shift with these choices, the overall qualitative results remain the same. Note that single scattering albedo is known to vary temporally and should be the subject of future investigations on how its dynamics influence soil moisture and VOD errors [53]. These values along with the true soil moisture, VOD pair were used to model the true TB values at each true soil moisture, VOD pair. Perturbations of soil moisture and VOD and their effects on the modeled TB were used to estimate the second derivative of J with respect to soil moisture or VOD.

Soil moisture and VOD experience increasing errors under differing scenarios of soil moisture and vegetation conditions: soil moisture becomes less robust with both greater canopy density (higher VOD) and greater soil moisture [see Fig. 2(A)]; VOD generally experiences greater error at higher mean VOD and under dry soil conditions [see Fig. 2(B)]. Note that some of the reduction of soil moisture and VOD error standard deviations at lower values is due to both variables having lower bounds.

In comparing the soil moisture and VOD error standard deviations across the estimation space, we investigated whether errors propagate more into VOD or soil moisture across a range of conditions. A metric to compare the relative robustness of soil moisture and VOD to error in a normalized fashion is the difference in their signal to noise ratios (SNR)

(5) $$\text{SNR Difference}=\frac{\sigma {\left(\text{SM}\right)}_{\text{Signal}}}{\sigma {\left(\text{SM}\right)}_{\text{Error}}}-\frac{\sigma {\left(\text{VOD}\right)}_{\text{Signal}}}{\sigma {\left(\text{VOD}\right)}_{\text{Error}}}$$

where we define *σ*(SM)_Signal_ and *σ*(VOD)_Signal_ as the standard deviation of the SM and VOD time series, respectively. SNR difference values greater than zero indicate more robustness of soil moisture to error and that measurement error is propagating relatively more into VOD.

While we can estimate the error standard deviations theoretically from (2), the true soil moisture and VOD signals are unknown. Making inferences about the true VOD and soil moisture signals is complicated by VOD and soil moisture having different units and dynamic ranges. To estimate the soil moisture and VOD signals and thus the SNR difference, we used dual channel algorithm retrievals of soil moisture and VOD from the SMAP and SMOS missions. These include 36 km gridded retrievals from the SMAP L1C TB descending measurements and SMOS L2 TB ascending measurements at 6AM between April 1st, 2015 and March 31st, 2018 [1], [54]. Since standard deviations of the full three-year time series were computed, each 36 km grid cell across global land surfaces will have an estimate of the SNR difference. We specifically estimated *σ*(SM)_Signal_ and *σ*(VOD)_Signal_ by taking the standard deviation of the deseasoned soil moisture and VOD time series. We estimated deseasoned soil moisture and VOD time series by computing a 30-day moving average window from the respective time series then subtracting it from the raw time series. This normalization removes the effect of large seasonal cycles in some regions; these regions would have a large signal estimate only due to a disproportionately large seasonal amplitude, not because of more robustness to error. We used the same DCA retrievals as in the experiments in Section IV.

**Fig. 3.**
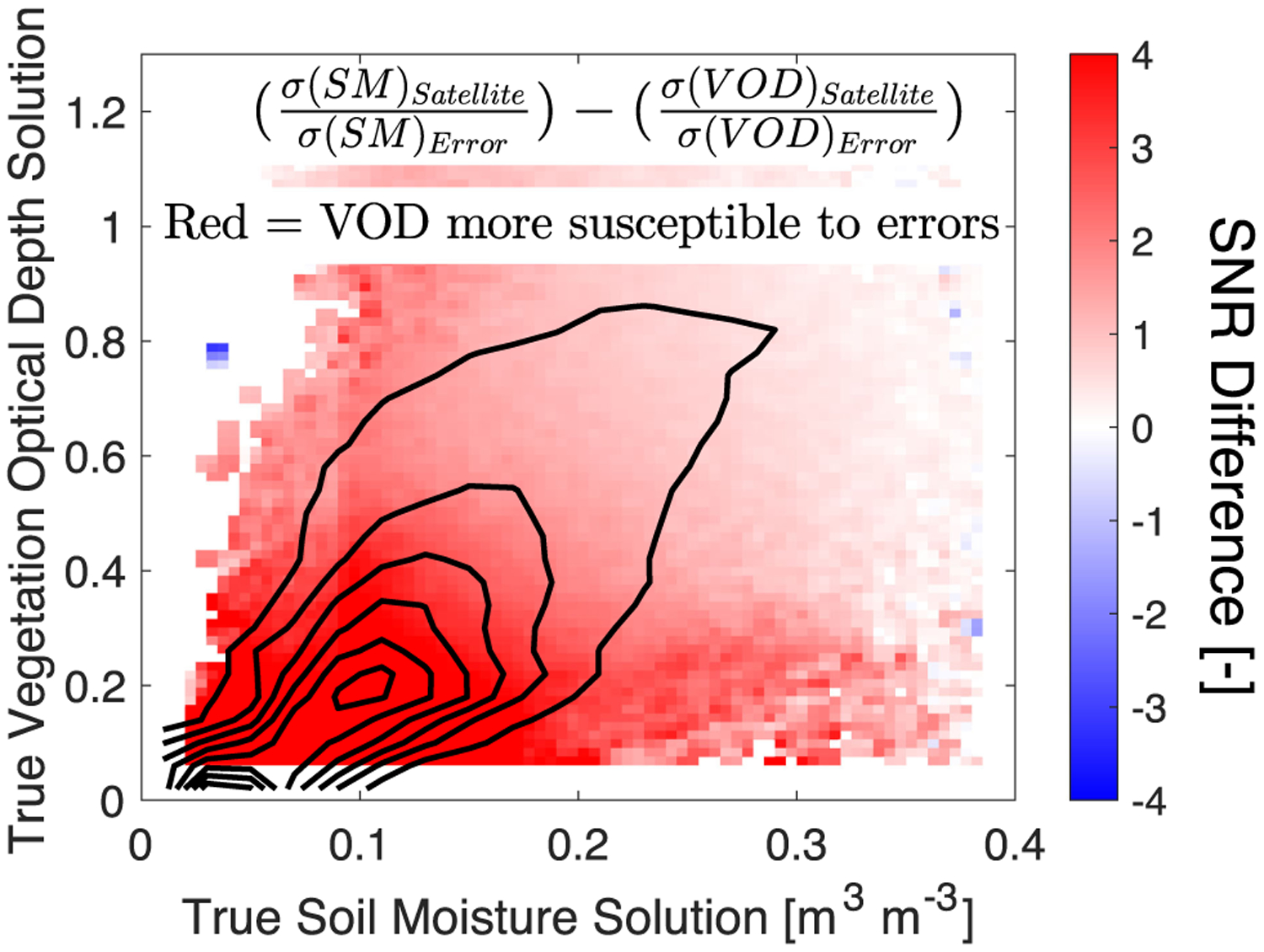
Degree to which errors propagate into VOD relative to soil moisture. Estimate of the SNR difference in (5) using satellite retrievals where each global 36 km land pixel includes a value plotted on this joint density. Values greater than zero indicate that error propagates into VOD more than soil moisture. Black contours show the joint density of SMAP time-mean values of soil moisture and VOD, and thus denotes locations in this space relevant to commonly observed conditions for the globe.

Assuming that the deseasoned standard deviation of SMAP VOD and soil moisture retrievals are good representations of the signal, error propagates into VOD relatively more than soil moisture for more than 99% of global land surfaces (see Fig. 3). The pattern estimated with SMOS is nearly identical (not shown). Over most of the globe, the SNR difference is between two and four meaning that the VOD signal would need to be an additional factor of two to four higher than that estimated by the satellite for the error to instead propagate into soil moisture (where the SNR difference would be negative). The SNR difference tends to be greatest over the commonly occurring global surface conditions (lower mean soil moisture and VOD). Conversely, regions with higher mean soil moisture and VOD inherently have lower sensitivity to soil moisture due to attenuation of microwaves through a dense canopy; their SNR differences are closer to zero meaning similar error propagation into soil moisture and VOD. Despite uncertain signal estimates, SNR differences commonly near four suggests a high degree of propagation of error into VOD relative to soil moisture. It also suggests that the overall finding is robust because it is unlikely that the satellite instrument and/or retrieval algorithm are underestimating true VOD variability by a factor of four.

Consider that without estimates of the true soil moisture and VOD signals as in Fig. 3, the ratio of Hessian matrix-estimated soil moisture and VOD error standard deviations in Fig. 2 can be used to establish the conditions for error propagation relatively more into VOD. Based on (5), the condition for error to propagate into VOD relatively more (SNR Difference > 0) can be written as

(6) $$\frac{\sigma {\left(\text{VOD}\right)}_{\text{Signal}}}{\sigma {\left(\text{SM}\right)}_{\text{Signal}}}<\frac{\sigma {\left(\text{VOD}\right)}_{\text{Error}}}{\sigma {\left(\text{SM}\right)}_{\text{Error}}}.$$

Equation (6) states that for error to propagate more into VOD, the ratio of the true VOD and soil moisture signals needs to be less than the ratio of their errors. For the inequality in (6) to hold and based on error ratio estimates from Fig. 2, the VOD signal would need to be generally less than three times the soil moisture variability across soil moisture and VOD conditions. Though the range of retrieved VOD magnitudes can extend to more than three times soil moisture’s range, retrievals of SMAP and SMOS instead show that VOD standard deviations are only a factor of 1.5 to 2 higher than soil moisture standard deviations for the majority of vegetated pixels. This is generally well below the ratios of the errors suggesting that the SNR difference is typically greater than zero across the globe.

## III. Effect of Regularization on Soil Moisture and VOD Retrieval Errors

We next investigate the effect of regularization on the retrieval errors compared to the nonregularized simultaneous retrieval approaches. Given the relatively higher VOD error sensitivity, one can anticipate reductions in VOD error when implementing a regularization approach. In this article, we regularized VOD using the MT-DCA, which retrieves VOD by constraining its dynamics in using TB information over a discrete number of satellite overpasses (two by default)

(7) $$\underset{X=\text{VOD},{\text{SM}}_{1},{\text{SM}}_{2}}{\min}J=\overset{2}{\underset{t=1}{\sum }}\underset{p=H,V}{\sum }{\left({\text{TB}}_{p\;\text{Obs}}(X)-{\text{TB}}_{p\;\text{Mod}}\right)}^{2}$$

where a single VOD and two soil moisture values are retrieved for an overpass pair using a total of four TB measurements. Equation (7) generates two time series of VOD: one where a given overpasses’ VOD is retrieved using TB information from the overpass before and one with the overpass after. This results in two VOD retrievals for each overpass. Both series are averaged. Thus, while VOD is held constant over a given overpass pair, this is repeated for multiple time-adjacent overpass such that the VOD is only temporally constrained for a given overpasses and not held constant. This is similar to the Sobolev-norm approach, which constrains the VOD time derivative between overpasses explicitly within the cost function [38], [55]. We note that our results are not specific to the MT-DCA here because repeating the analysis with the Sobolev-norm regularization provides the same qualitative findings (not shown). These approaches are different from the Tikhonov regularization approach, which constrains deviations from an *a priori* input time series of VOD [39]. The MT-DCA is chosen here because it does not require an *a priori* guess of the VOD variations and the timescale of constraints between discrete overpasses is more tangible than the Sobolev-norm continuous-based timescale representation [33], [38]. Results here are most applicable to the MT-DCA and Sobolev-norm regularization approaches. The results apply to the Tikhonov approach, but Tikhonov approach would additionally be a function of the chosen input *a priori* VOD time series. Comparison between the retrievals of MT-DCA and other regularization approaches is beyond the scope of this article.

There are two major assumptions of this time-constrained regularization. First, the true VOD behavior must be slower in time (such as, more autocorrelated) than the soil moisture dynamics. If dynamics of soil moisture are slower, then soil moisture could instead be regularized to reduce errors. If the daily dynamics of both variables occur over similar timescales, regularization may not reduce errors. Nevertheless, such conditions of more rapid VOD dynamics than soil moisture are unlikely given known contribution of relatively slower dry biomass to VOD across timescales as well as VOD retrievals globally indicating slower responses to rain events than soil moisture [5], [7], [42]. Second, TB changes between overpasses are non-negligible. Insufficient changes in TB between overpasses fail to provide additional TB information and the optimization converges to that of the DCA. Such cases can occur if surface conditions remain stagnant or the instrument sampling frequency is too small to observe surface changes. However, under most conditions, surface conditions change sufficiently between the 1–3-day SMAP and SMOS satellite revisits used in this article.

To first assess the effect of regularization on soil moisture and VOD errors, we ran experiments where we simulated true soil moisture and VOD time series such that the true soil moisture and VOD solutions are known and algorithm performance with respect to the true solutions can be assessed. We then converted these time series into TB using the tau–omega model and obscured the original signal by adding random noise. The same noisy TB simulated time series are input into each algorithm (regularized and simultaneous) and retrieved soil moisture and VOD are produced. We then computed error deviations from defined “true” time series using algorithms with and without VOD regularization in the presence of random noise. No algorithm has an advantage over another because they all receive the same noisy inputs. The difference between the error deviations from the regularized and simultaneous retrieval algorithms isolates only changes in error due to regularization. Comparison between different satellite-based products would instead be additionally a function of algorithmic parameterization choices and sources of TB observations [38].

**Fig. 4.**
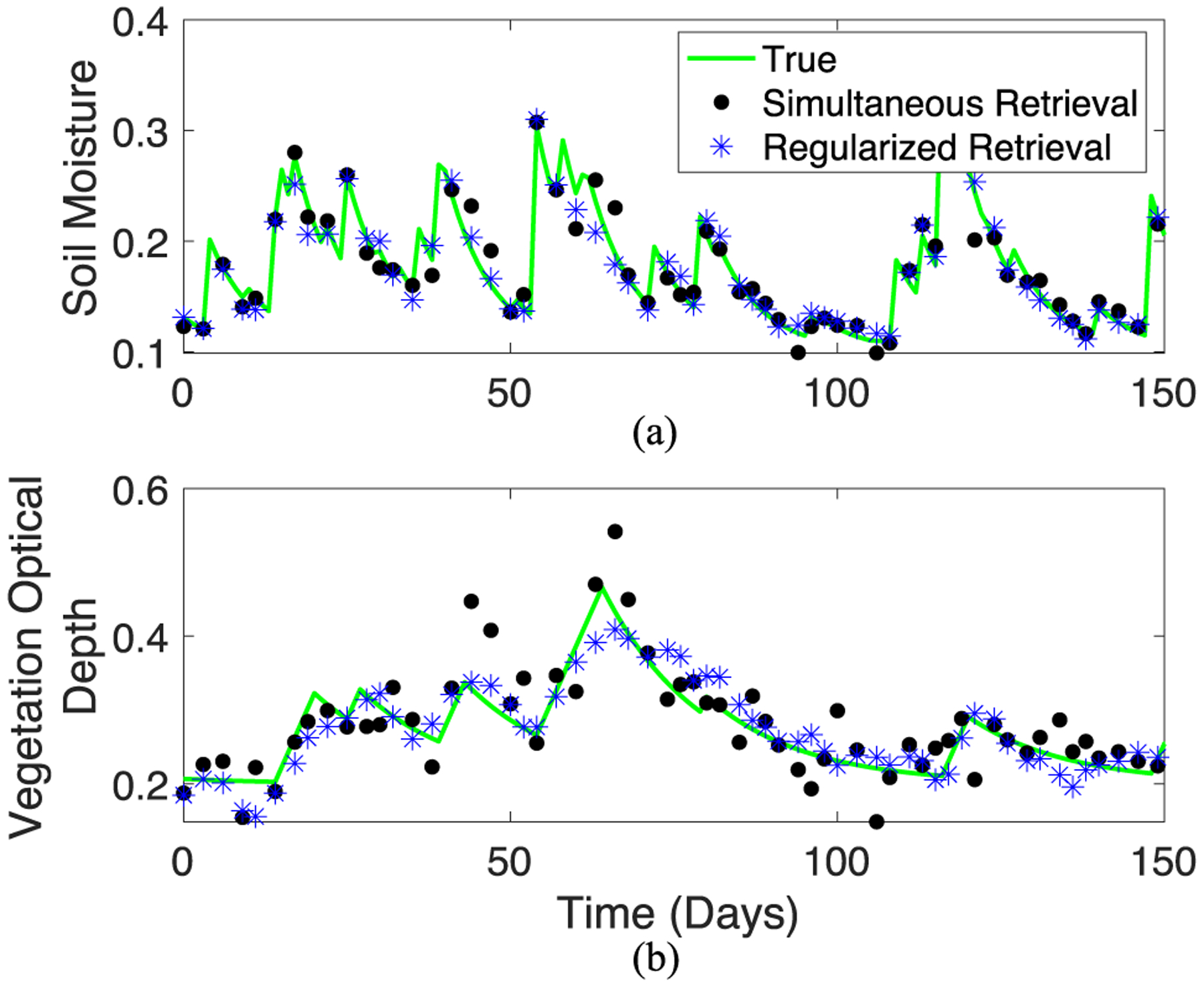
Example simulated time series for (A) soil moisture and (B) VOD truth, retrieved without regularization (DCA), and retrieved with VOD regularization (MT-DCA). Simulated time series with retrievals given a 1.1K TB error standard deviation.

Here, true soil moisture time series were simulated using a stochastic rainfall generator: precipitation was randomly generated based on random draws of daily probability of rainfall and storm intensity from uniform and exponential distributions, respectively. The simulated precipitation time series was then translated into soil moisture with gains based on a water balance “bucket” model and losses based on exponential losses empirically determined previously [56]. True vegetation optical depth time series were then generated that physically depend on the soil moisture time series. Specifically, after rainfall, higher soil moisture values result in increasing VOD and lower soil moisture values result in decreasing VOD as found in previous studies [7]. In addition to lower frequency biomass climatology contributions, such VOD variations include higher frequency variability on sub-weekly timescales given expected contributions from daily water loss, rehydration, and known rapid growth dynamics in semiarid biomes [16], [42], [45]. This results in lagged VOD responses to soil moisture variations as identified previously in both simultaneous and regularized VOD retrievals [42], [57]. Example true time series are shown in Fig. 4. We examined the limiting case of VOD exactly varying with soil moisture; such a scenario can occur if VOD is only a function of water potential in the canopy, which generally varies exactly with soil water potential at predawn. In violating one of the main assumptions of regularization, this scenario generally leads to converging behavior between the DCA and MT-DCA as expected (not shown). Ultimately, the purpose is for the synthetic time series to resemble observed satellite soil moisture and VOD so that the results are applicable to the VOD behavior retrieved by satellites. We assert that simulating the exact *in situ* dynamics is not necessary to address our research questions here because a range of selected dynamics would describe emerging qualitative behavior from the radiative transfer model and algorithm. The magnitudes of error metrics should be interpreted with caution because we anticipate some dependence on the chosen true VOD dynamics and parameter selections. Nevertheless, the VOD scenarios here likely present conservative estimates of regularization’s error reduction in emphasizing rapid, subweekly contributions to VOD temporal dynamics.

Next, the soil moisture and VOD time series were used to create synthetic, true TB_H_ and TB_V_ time series by forward modeling with the tau–omega model. Randomly generated normally distributed noise on the order of that determined for the SMAP satellite [e.g., N(01.1K)] were then added to these true TB series. Finally, using these noisy TB time series, soil moisture, and VOD were estimated using the tau–omega model within both DCA (VOD without regularization) and MT-DCA (VOD with regularization) algorithms, while holding all other radiative transfer parameters constant. See Fig. 4 for noisy retrieved time series examples based on the simulated TB time series. The root-mean-square error (RMSE) is computed between the true simulated series and retrieved series both for soil moisture and VOD. The default MT-DCA algorithm was used which constrains VOD dynamics between time-adjacent overpasses (1–3 days). This procedure was repeated in a Monte Carlo setting by randomly generating TB noise [e.g., N(0, 1.1K)] to generate soil moisture and VOD error distributions. The same parameters as in Figs. 2 and 3 were used here. Note that the TB time series were converted to emissivity by normalizing by physical temperature such that physical temperature variations and underlying assumptions are factored out.

**Fig. 5.**
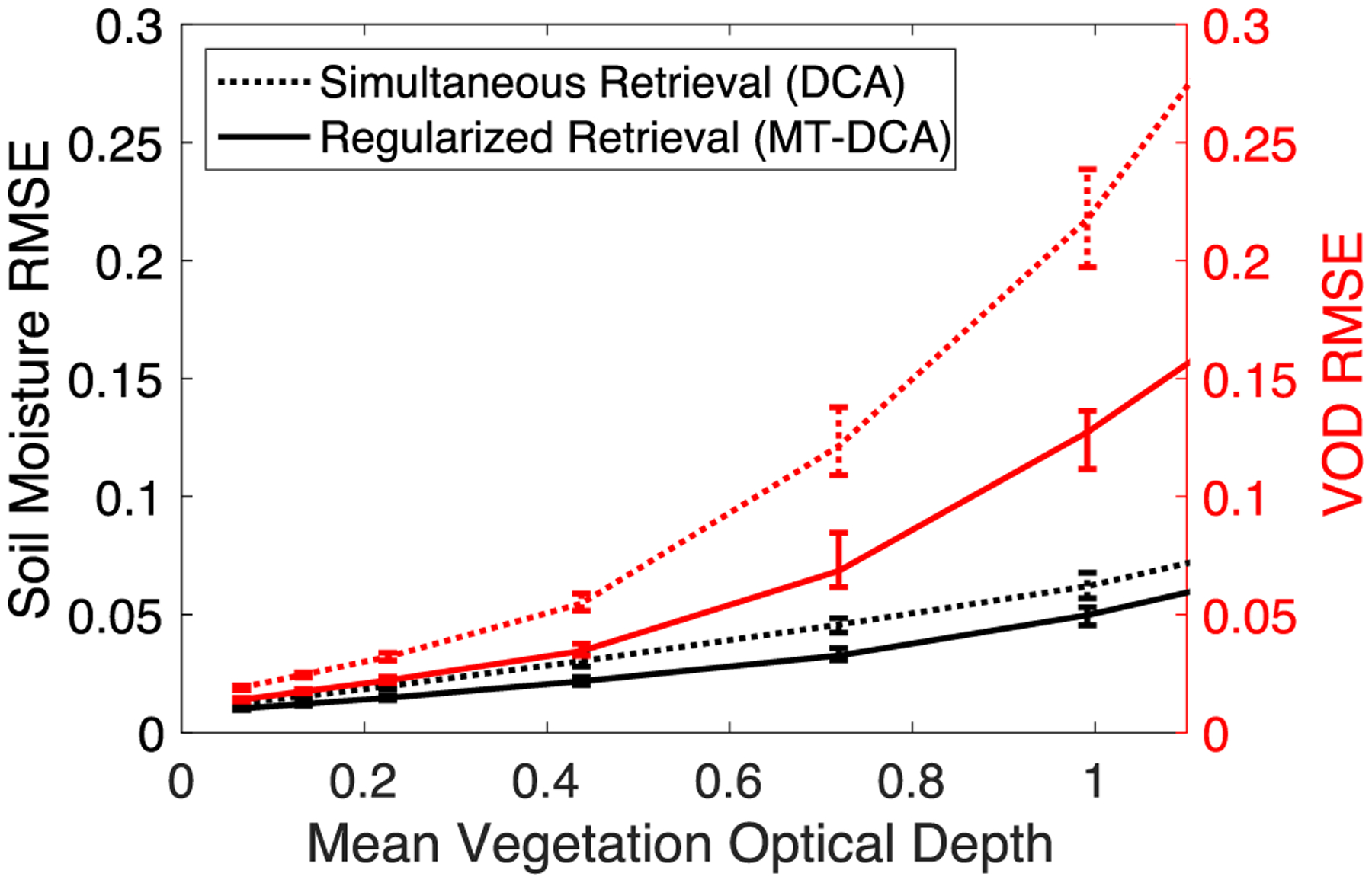
Regularization reduces both soil moisture and VOD errors. RMSE of soil moisture and VOD for simultaneous retrieval algorithm (DCA) and VOD regularization algorithm (MT-DCA). Computed based on simulated time series where retrievals with random noise inputs were compared with true simulated soil moisture and VOD time series. Error bars show 95% confidence interval. Computed given a 1.1K TB error standard deviation and average soil moisture of 0.2 m^3^ m^−3^.

The aforementioned simulation procedure isolates VOD and soil moisture’s response to measurement noise as it propagates through each algorithm. It also isolates the effect of regularization on a given variable’s errors in comparing errors from the DCA and MT-DCA. As expected, the soil moisture and VOD RMSE from the simultaneous retrieval algorithm (DCA) within these simulations have similar magnitudes as error standard deviations computed theoretically in Fig. 2 (compare Figs. 2 and 5).

Across a range of scenarios in varying mean soil moisture and mean VOD, VOD RMSE are reduced by an average of 36% when using regularization (see Figs. 5 and S1). Soil moisture RMSE are also reduced by an average of 22% with regularization (see Fig. 5). This is less than that for VOD which is a consequence of soil moisture’s already larger robustness to error (see Fig. 3). Nevertheless, it has been shown previously that VOD regularization brings satellite soil moisture dynamics closer to *in situ* observed dynamics [27], [33], [38]. It can, thus, be expected that regularization will create greater corroboration with independent datasets for VOD than that found for soil moisture. Equivalent studies for VOD are becoming possible with more ground vegetation measurements of VOD or observationally-constrained crop model output related to VOD [15], [18], [19]. However, a greater number of sites across land cover types will be needed to obtain robust results. Ideally, measurements of VOD from multiple tower radiometers within a satellite footprint would provide the most direct comparison with satellite VOD, though such an effort is cost restrictive. Another method is to use measurements of percent saturation and dry biomass at satellite footprint scales (i.e., 36 km) along with a method to convert these parameters to VOD, as in a recent study [18]. We nevertheless provide further simulation-based and indirect observation-based support for this expectation in Section IV.

An example of the aforementioned effects can be seen in Fig. 4. Error propagates more into VOD than soil moisture as seen in the greater departures of the simultaneous retrievals for VOD from the true values than for soil moisture. Such large VOD departures from truth have been shown in previous simulations [38]. For example, simultaneous VOD retrievals can result in greater false detection of VOD increases when only VOD loss is detected (see Fig. 4(B) between days 70 and 110). Regularized VOD retrievals reduce these VOD departures and are better able to detect peaks and declines in VOD on average [see Fig. 4(B)].

Greater regularization can be applied with VOD constrained over longer timescales, analogously to increased regularization in the Sobolev-norm approach. We repeated the MT-DCA retrieval process for the conditions in Fig. 5, but with VOD constrained and measurements used for retrieval over three time-adjacent overpasses (4–6 day duration because of two satellite overpass intervals) instead of two overpasses. We found that there are diminishing benefits: on average VOD RMSE are only reduced by an additional 8% and soil moisture RMSE by an additional 2% beyond that of the error reductions from the default level of regularization in the original MT-DCA (see Figs. S1 and S2). Furthermore, increasing the degree of VOD regularization can result in obscuring VOD dynamics that are known to occur on subweekly timescales [42] (see Section IV).

**Fig. 6.**
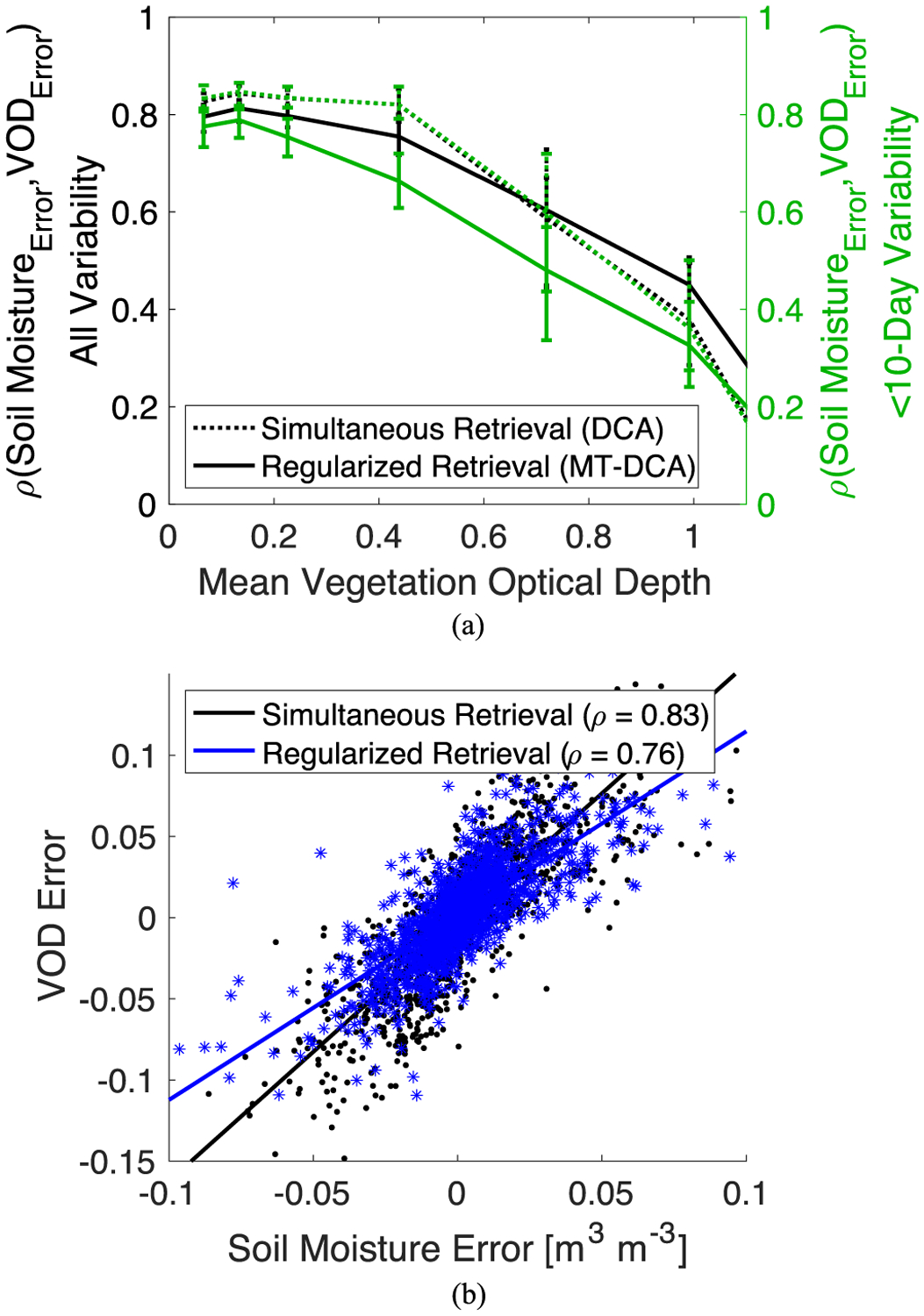
While soil moisture-VOD errors are positively correlated, regularization tends to reduce this coupling, especially at short timescales. Temporal correlation between soil moisture and vegetation optical depth errors. Shown for both the simultaneous retrieval algorithm (DCA) and VOD regularization algorithm (MT-DCA). (A) Pearson correlation coefficient between the errors. The right axis (green lines) shows the error correlation when only considering variability of less than 10-days. (B) Example raw soil moisture-VOD error relationship from same simulation as that shown in Fig. 4 (p < 0.05). Computed based on simulated time series where retrievals with random noise inputs were compared with true simulated soil moisture and VOD time series. Error bars show 95% confidence interval. Computed given a 1.1K TB error standard deviation and average soil moisture of 0.2 m^3^ m^−3^.

Another benefit of regularization is that it reduces the positive relationship between soil moisture and VOD errors (see Fig. 6). Here, errors are computed as the difference between the retrieved and simulated true values for each time step. Because of the cost function structure generated by the tau-omega model (see Fig. 1 in [37]), soil moisture and VOD errors are positively related (see Fig. 6). Errors propagating through the estimation move along a valley within the cost function. Such an effect is amplified when using a simultaneous retrieval approach such as the DCA or LPRM that is ill-posed due to correlated TB measurements. Note that at high VOD the valley within the cost function orients along the VOD axis and the spurious positive soil moisture-VOD coupling inherently reduces (see Fig. 6). Nevertheless, this relationship prescribed within the tau–omega model can confound investigations of soil-plant relations because it does not reflect real soil moisture-VOD coupling. In reducing soil moisture and VOD’s sensitivity to noise, regularization reduces the correlation between soil moisture and VOD errors at lower mean VOD and across conditions at higher frequency components of variability for soil moisture and VOD (see Fig. 6). This effect propagates into reduced spurious correlations between the soil moisture and VOD signals (see Section IV and [33]). Greater regularization reduces the correlation further, but again at the expense of obscuring real VOD dynamics on short timescales (see Section IV).

## IV. Effect of Regularization on the VOD Signal

Though regularization reduces the VOD signal’s errors and spurious coupling with soil moisture, it is unclear whether regularizing VOD will obscure real VOD dynamics at the shortest timescales observed. We first evaluate the impact of regularization on satellite VOD retrievals in comparing VOD with and without regularization holding all other parameters constant. Specifically, we compare nonregularized and regularized VOD retrievals from DCA and MT-DCA approaches, respectively, using SMAP L1C TB measurements between April 1st, 2015 and March 31st, 2019 [54], [58].

We choose this approach for several reasons. First, we were able to apply the DCA and MT-DCA with the same radiative transfer parameters such that the only difference between the algorithms’ retrievals is that regularization is applied in the MT-DCA. It, therefore, isolates only the effect of regularization on retrieved soil moisture and VOD rather than other algorithmic decisions or data sources. We are unable to conduct such a controlled experiment with official SMAP or SMOS products. Second, in using our own algorithmic framework, the same approach applied to the synthetic data in Figs. 4 to 6 can be applied to the observed satellite TB data as well. This minimizes differences in algorithm frameworks used throughout the study. Third, the MT-DCA regularization was again used instead of the Sobolev approach because of simplicity in interpreting how the VOD regularization was applied (see Section III). Nevertheless, these results are sensitive only to the fundamental structure of the tau–omega model and effects of regularization—they are not sensitive to the choice of the algorithm or product. Thus, the same qualitative results would be found using alternative algorithms and regularization approaches. Our goal is to evaluate differences in regularized and simultaneous retrievals, not to compare regularization approaches or satellite products. We are not arguing in favor of any of the dataset sources or algorithms presented here.

To test the effects of regularization on different VOD frequency spectra, we computed the power spectral density of the difference between global satellite retrieved MT-DCA VOD and DCA VOD. The Lomb-Scargle periodogram method was used due to irregular sampling frequencies in the microwave satellite overpasses [59].

**Fig. 7.**
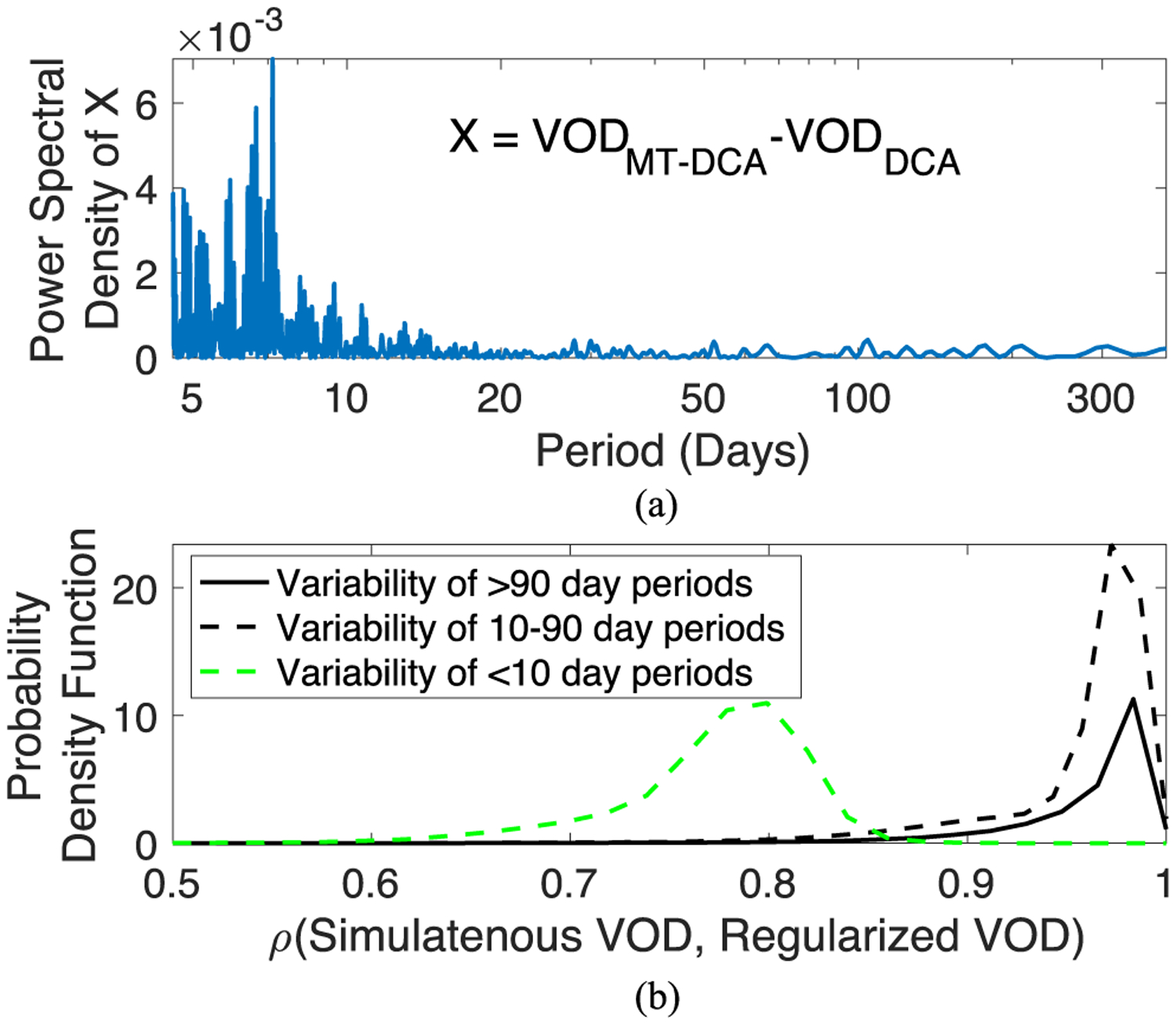
Simultaneous (DCA) and regularized (MT-DCA) VOD retrievals tend to differ most at higher frequencies (<10-day variability). (A) Power spectral density of difference between regularized and simultaneously retrieved VOD. Shown using SMAP data for a representative grassland pixel in Chad (13.7°N, 16.0°E). (B) Probability density function of correlation between DCA VOD and MT-DCA VOD for different spectra using SMAP satellite retrievals from all vegetated regions across the globe.

We find that regularized VOD appears to show differences from nonregularized VOD mainly for periods of variability below 10 days [see Fig. 7(A)]. This is expected given that regularized VOD is constrained over 2-to-3-day periods and spurious coupling reductions due to regularization appear largest at this timescale (see Fig. 6). Thus, this would mainly influence VOD’s subweekly variations.

We further investigate here whether regularization influences only high frequency VOD variability across the globe. We define high frequency here as the shorter timescale dynamics observed for VOD of periods less than 10 days. We isolated DCA and MT-DCA variability below a 10-day threshold chosen based on Fig. 7(A). We suspected that there could be additional effects on subseasonal variability up to 90 days. Therefore, the low frequency (periods>90 days) and two high frequency bands (periods<10 days and 10–90 days) of VOD were partitioned from both the DCA and MT-DCA retrievals and then compared. At each location, 10- and 90-day moving average windows were fit to each VOD time series to approximately decompose each frequency band for each pixel across the globe. The 90-day moving average window produces time series with only low frequency variability. The 10-day moving average was subtracted from the VOD time series to produce a time series with high frequency variability of approximately less than 10-day periods. Finally, 10–90-day variability was computed by subtracting both the greater than 90-day moving average and 10-day high frequency variability from the VOD time series. This process was repeated for both the DCA and MT-DCA at each pixel. We computed Pearson’s correlation coefficients between the MT-DCA and DCA VOD variability within each spectral band to determine the effect of regularization on these components of the signal.

Across the globe, regularizing VOD mainly influences the high frequency VOD dynamics below 10 days, reducing temporal correspondence with the nonregularized retrievals [global mean *ρ* = 0.77; global mean absolute mean difference < 0.001; Fig. 7(B)]. The lower frequency seasonal and interannual dynamics as well as 10–90-day subseasonal dynamics are less impacted [global mean *ρ* > 0.95; Fig. 7(B)].

We caution that Fig. 7 does not suggest that regulation should only be applied when interpreting subweekly VOD variations. Rather, Fig. 7 serves as motivation to further evaluate the subweekly VOD signal, which changes most with regularization. It is well known that regularization reduces soil moisture errors [24], [33]. Furthermore, analyses evaluating only low frequency properties of VOD (i.e., seasonal cycles, annual means, etc.) are still impacted by high frequency noise and would have error reduction benefits from regularization. This is because the high frequency variability due to noise has large impacts on the RMSE of the full VOD time series (see Figs. 5 and 6). Even the low frequency VOD variability alone shows root mean square differences of 0.01 and mean absolute biases of 0.01 (increasing to 0.03 in forested regions) between simultaneous and regularized low frequency VOD retrievals. It was also recently shown that the spurious coupling between soil moisture and VOD persists on monthly timescales, which can be reduced with regularization [33]. Therefore, regularization can remove effects of noise holistically in soil moisture and VOD time series.

While regularization influences high frequency variability of VOD relatively more, it is unclear whether regularization benefits short term VOD dynamics in removing errors or obscures real VOD dynamics in applying temporal constraints. We first assess this issue using our simulations. Here, we correlated the noisy retrievals (with and without regularization) with the true simulated time series at high frequencies (periods <10 days). We also evaluated how much soil moisture-VOD correlation, a coupling metric, deviates from the true simulated coupling with and without regularization. Such an investigation can isolate whether regularization improves satellite VOD’s ability to represent true VOD dynamics at high frequencies, the subject of recent work [11], [42].

**Fig. 8.**
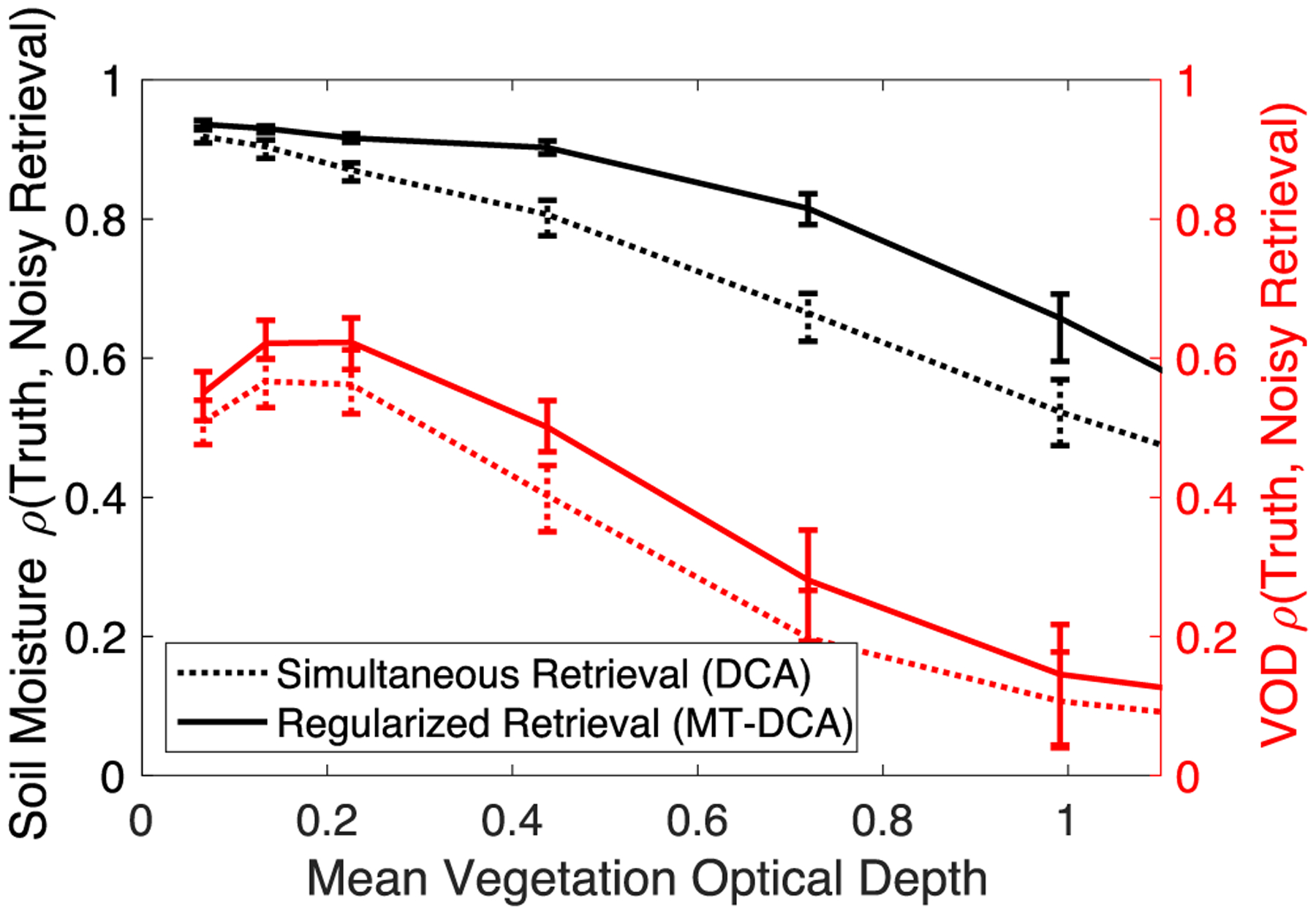
Regularization increases soil moisture and VOD temporal correlation with true variations on sub-weekly timescales. Correlation between simulated noisy time series and truth for soil moisture and VOD for their high frequency components (<10 day periods). Shown for both the simultaneous retrieval algorithm (DCA) and VOD regularization algorithm (MT-DCA). In all cases, differences between DCA and MT-DCA metrics are statistically significant (p < 0.01). Computed based on simulated time series where retrievals with random noise inputs were compared with true simulated soil moisture and VOD time series. Computed given a 1.1K TB error standard deviation and average soil moisture of 0.2 m^3^ m^−3^.

**Fig. 9.**
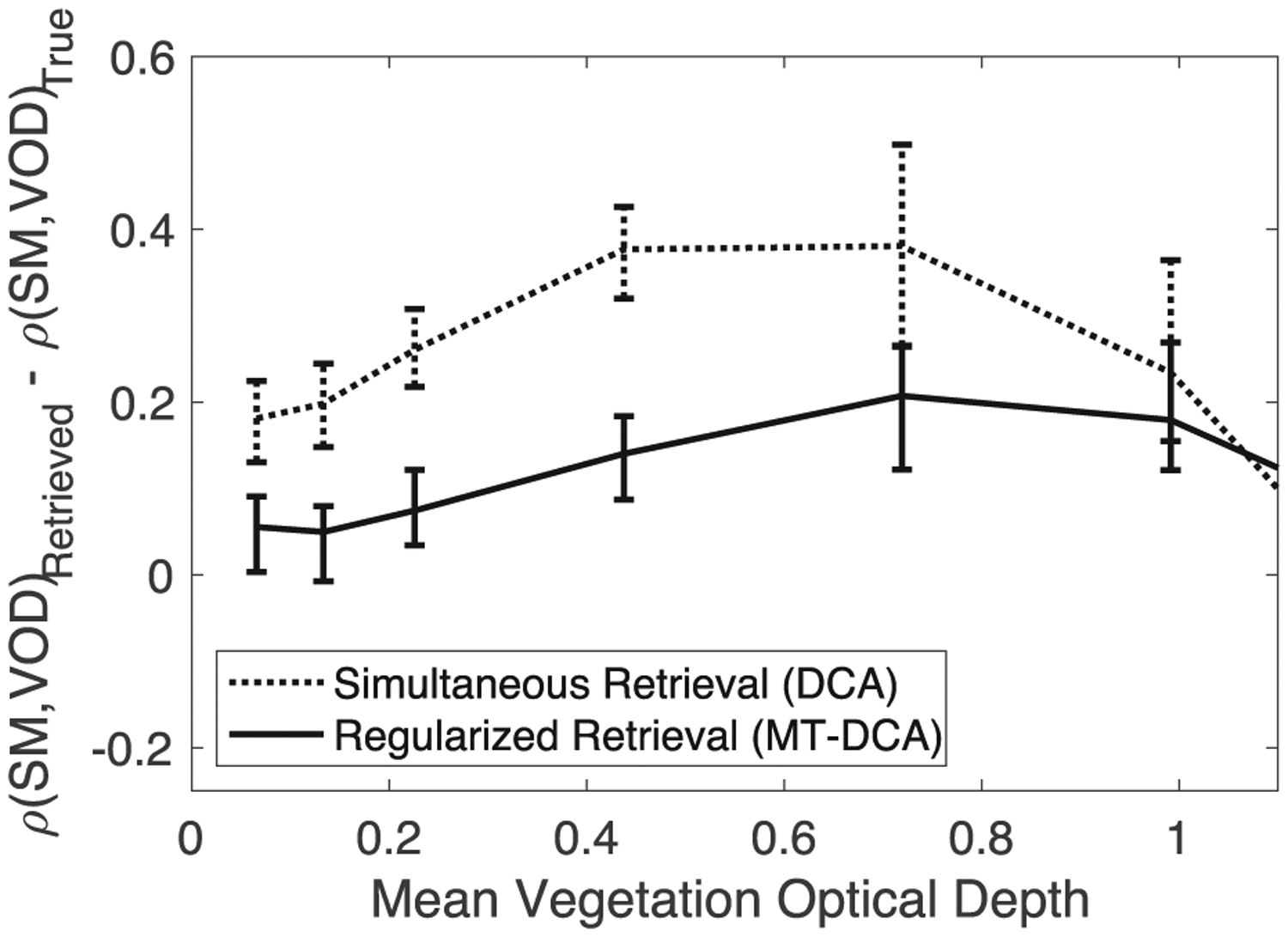
While simultaneous retrieval methods positively bias soil moisture-VOD coupling, regularization reduces this bias. Difference between the retrieved soil moisture-VOD correlation and the true correlation for their high frequency components (<10-day periods). Shown for both the simultaneous retrieval algorithm (DCA) and VOD regularization algorithm (MT-DCA). Note that the correlations are positive in nearly all cases. Computed based on simulated time series where retrievals with random noise inputs were compared with true simulated soil moisture and VOD time series. Computed given a 1.1K TB error standard deviation and average soil moisture of 0.2 m^3^ m^−3^.

We find that regularized retrievals more closely resemble the true high frequency variability of both soil moisture and VOD (see Fig. 8). Across scenarios, regularization significantly (p < 0.01) increases the correlation of VOD with true dynamics at high frequencies (periods of <10 days). Statistical significance was determined based on parametric two sample t-tests of means (where Kolmogorov–Smirnov tests did not indicate non-normal distributions) [60]. Regularization of VOD additionally increases the soil moisture correspondence to truth at subweekly timescales (see Fig. 8). Regularized retrievals also more closely resemble the true soil moisture-VOD coupling (see Fig. 9). The regularized retrieval’s improved resemblance to the truth is largely because the spurious positive correlations due to error are reduced (see Fig. 6). This error correlation tends to positively bias correlation metrics evaluating soil moisture-VOD coupling mostly at high frequencies (see Fig. 9). Note that the exact magnitudes of correlation depend on the temporal dynamics of the simulated soil moisture and VOD time series. The qualitative result that holds across conditions is that regularization reduces these error correlations such that soil moisture-VOD coupling can be more reliably evaluated (see Fig. 9). The reductions in spurious coupling translate to lower frequency components of the soil moisture-VOD time series as shown previously [33]. Therefore, recent studies evaluating soil moisture and VOD dynamics across timescales, and especially shorter timescales (i.e., drydown dynamics), can benefit from regularization [56], [61]. Note that the ability to represent true VOD high frequency dynamics under heavy tree cover (high VOD) is generally lower altogether (see Figs. 5 and 8).

Increasing the degree of regularization by constraining VOD over more overpasses presents diminishing returns. In constraining VOD over three instead of two overpasses, the full VOD signal experiences marginal, nonstatistically significant mean increases in correspondence between truth and retrieved VOD in the presence of noise (see Fig. S2). However, under greater regularization, the higher frequency, subweekly VOD variability tends to show decreases in correspondence between the retrieved VOD series and truth, especially for lower mean VOD (see Fig. S3). This is expected given that regularization mostly imposes restrictions on VOD changes on shorter timescales. Over-regularizing will thus obscure real VOD changes on subweekly timescales in taking on features of a low-pass filter. Given the timescale of physical processes governing VOD changes and satellite overpass revisit timescale, it appears that regularizing VOD over periods of no more than three days is optimal, especially for regions with lower mean VOD (see Fig. S3). Some biomes like forested regions may have a different optimal degree of regularization. In the case of the Sobolev-norm regularization approach, the parameter penalizing changes in VOD would need to be similarly adjusted to determine at what point over-regularization occurs as in [33].

Do the effects and perceived benefits of regularization appear in global VOD responses to climate, especially at shorter timescales? We evaluated the satellite observed VOD response to rainfall with and without regularization to assess imprints of errors and regularization found in the simulations on the satellite-retrieved VOD subweekly variability. We computed the percentage of rainfall events where VOD increases (likely representing plant water uptake faster than transpiration or biomass loss) for both the DCA and MT-DCA. Rainfall events were defined using the soil moisture drydown identification technique in [42].

**Fig. 10.**
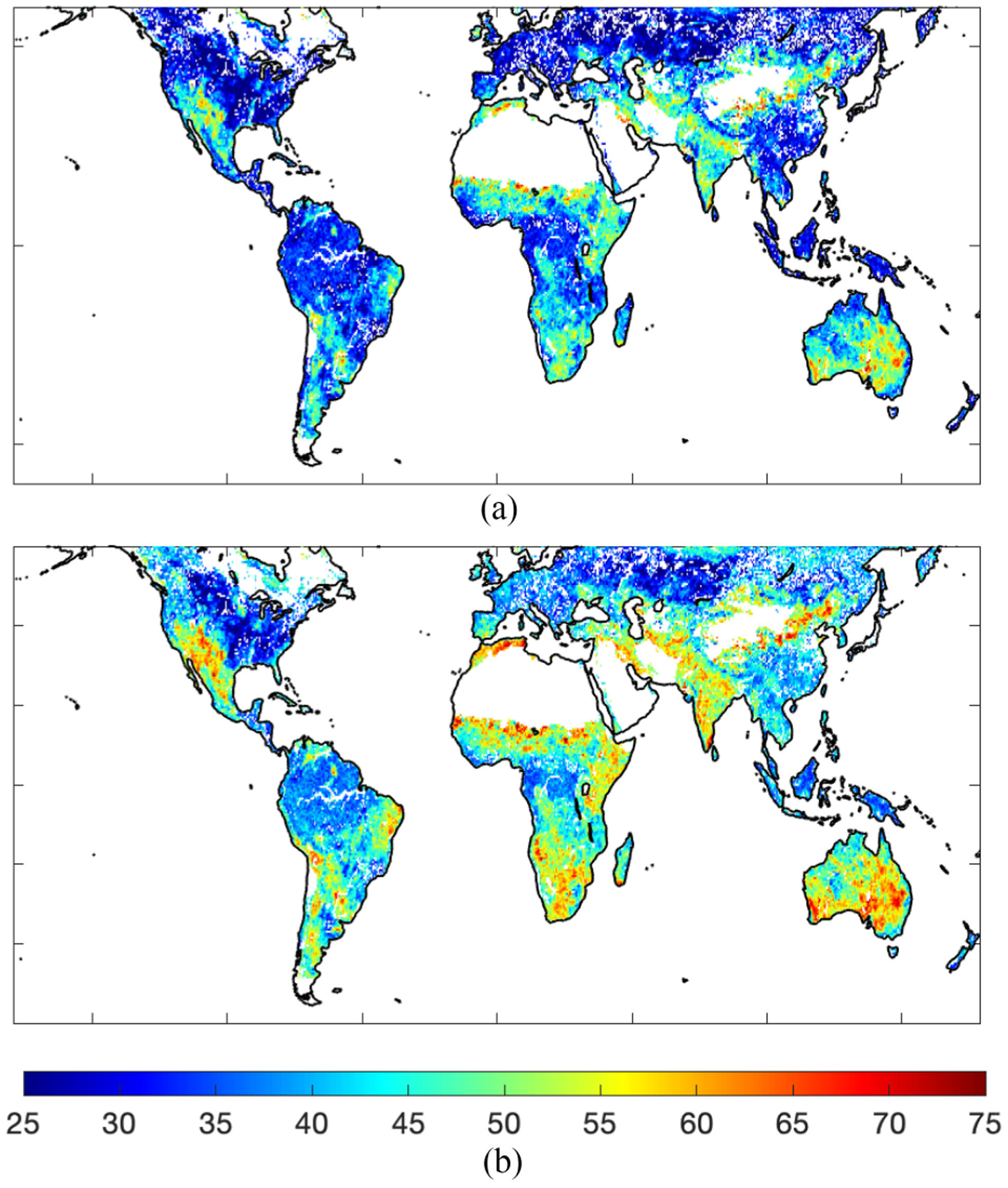
Reduced spurious soil moisture-VOD coupling from regularization results in more frequent VOD increases after rain events as shown in global retrievals. Plotted variable shows percentage of storms where there is a VOD increase response during soil drying on the first overpass after a storm.Vegetation optical depth post rain storm response from (A) dual channel algorithm and from a (B) regularization approach (MT-DCA). Computed based on VOD retrievals from SMAP TB measurements.

VOD dynamics on subweekly timescales, in response to storm events, show the same global spatial pattern between the two algorithms, but with a mean bias (see Fig. 10). Specifically, as found previously, SMAP VOD shows more frequent increases after rainfall in semiarid regions than in more humid, wooded regions across both algorithms (see Fig. 10) [7], [42]. This response and spatial pattern are shown in both regularized and nonregularized VOD retrievals (spatial Pearson correlation coefficient of 0.89; p<0.01). However, a mean bias is present: MT-DCA VOD increases in response to storms occur more frequently (by 7% on average) than VOD increases from the DCA.

We argue that the DCA having less frequent VOD increase responses is due to measurement error artificially inducing a positive relationship between soil moisture and VOD (see Figs. 6 and 9). Since the VOD responses are evaluated during soil moisture drying (decreases) after storms, measurement error will tend to cause VOD to decrease in step with soil moisture artificially, as shown in Fig. 6. Thus, regardless of the true VOD response during soil drying, noise will negatively bias the metric in Fig. 10 in driving VOD decreases. Thus, with greater susceptibility to error in the DCA, DCA-retrieved VOD artificially decreases more frequently after storms. In contrast, regularization within the MT-DCA reduces the positive soil moisture-VOD error relationship, as shown in Fig. 6, by reducing the correlation of soil moisture and VOD errors. As shown in Fig. 9, this reduces biases in soil moisture-VOD coupling. As such, regularized VOD shows more frequent increases after storms; poststorm VOD increases would be a sign of a negative soil moisture-VOD relationship. Furthermore, these regularized VOD increases are less prone to spurious changes due to noise (see Figs. 5, 8, and 9). These more frequent VOD increases, especially in semiarid regions, are expected from recent investigations linking these VOD responses to rapid plant hydraulic and photosynthetic mechanisms [42], [62]. A similar case of error correlations may be occurring in croplands where post-growing season artificial VOD increases manifest from improperly representing tilling-based soil roughness increases [18], [26].

Of note is that the regularized retrievals also still show rainfall responses indicating that the subweekly signal is not suppressed. If the MT-DCA regularization suppressed subweekly VOD dynamics as in a low-pass filter, the spatial pattern shown using the simultaneous retrievals (DCA) would not persist and the percentage of storms where VOD increases computed based on the regularized VOD (MT-DCA) would converge to 50% indicating no consistent response (with equal, random increases and decreases of VOD after storms where VOD is decoupled from rainfall driven soil moisture responses).

In summary, the regularized VOD poststorm behavior shown in the satellite retrievals likely reflects real VOD dynamics at subweekly timescales beyond that of snapshot DCA retrievals (see Fig. 10). First, regularization increases occurrence of VOD increases after storms, which is indicative of more negative soil moisture-VOD relationships. This suggests DCA retrievals are influenced by error-induced positive soil moisture-VOD relationships (see Fig. 6) and that regularization reduces these artifacts. Second, despite regularization potentially imposing constrained VOD change restrictions between overpasses that could obscure short-timescale VOD dynamics, we show that moderately regularized VOD can represent the true VOD dynamics at subweekly timescales more than simultaneous VOD retrievals can (see Figs. 8 and 9). Additional credence is given to the regularized post-rainfall VOD responses in Fig. 10(B) because they were previously found to reflect *in situ* plant hydraulic and photosynthetic responses [42], [62]. However, greater regularization instead begins to obscure the subweekly VOD signal (see Fig. S3), despite modest error reductions of the entire VOD signal. Nevertheless, there is no evidence that the VOD responses are subdued or overconstrained here in using the default MT-DCA that uses moderate regularization. This is because both regularized and simultaneous retrievals show similar spatial patterns of VOD response in Fig. 10, whereas over-regularized retrievals would converge to values of 50% everywhere (with random increases and decreases of VOD).

Ultimately, a moderate degree of regularization brings VOD closer to the simulated true VOD time series, improves soilmoisture VOD coupling metrics, and removes features of noise-based artifacts from the plant pulse-response rainfall responses compared to simultaneous VOD retrievals. Equivalent investigations should be attempted on how error patterns here influence studies evaluating diurnal changes in VOD [11], [63].

## V. Conclusion

We find that microwave sensor measurement errors tend to propagate disproportionately into vegetation optical depth (related to water mass per unit area in the vegetation canopy) compared to soil moisture. Measurement errors result in spurious relationships between soil moisture and VOD that confound their joint study, for example, of soil-plant water relations. Moderately constraining (or regularizing) VOD dynamics by using measurement information from temporally adjacent satellite overpasses creates greater soil moisture and VOD robustness to error and reduces spurious positive relationships between soil moisture and VOD. As such, regularized VOD is closer to the true simulated VOD variations and soil moisture-VOD coupling biases are reduced, especially at the shortest timescales observed. This results in reduced effects of noise on globally retrieved VOD rainfall responses, linked previously to plant rehydration and growth. We, therefore, recommend VOD time-constrained regularization for the large-scale study of vegetation temporal dynamics and soil-plant water relations across timescales. This advocates for recent efforts to regularize VOD in SMAP and SMOS official products. We also recommend further work in evaluating the optimal degree of regularization needed as more *in situ* vegetation information related to VOD becomes available at large scales. The results mainly pertain to time-derivative constraints such as in the MT-DCA and Sobolev-norm regularization approaches. Further work is needed to determine if inputting *a priori* VOD time series such as in the Tikhonov regularization confers the same benefits outlined here.

As these results reflect the formulation of the radiative transfer model, they may change with alternative electromagnetic modeling approaches of surface microwave emission (such as, first-order models that are applicable for woody vegetation or multifrequency approaches). However, the majority of soil moisture and VOD retrieval approaches maintain the general structure of the tau–omega model. These results are, thus, anticipated to be general and apply to retrieval approaches across microwave soil moisture satellite missions.

## Supplementary Material

supp1-3124857
