# Supplementary material for: Error Propagation in Microwave Soil Moisture and Vegetation Optical Depth Retrievals
Source: IEEE J Sel Top Appl Earth Obs Remote Sens. Author manuscript; Available in PMC 2022 Jan 7. (PMC8740529; doi:10.1109/jstars.2021.3124857)
Supplement: supp1-3124857 [file NIHMS1758108-supplement-supp1-3124857.docx]

Fig. S1. Percent reduction in error with regularization as compared to errors from simultaneous retrievals, where N equals the number of satellite overpasses that VOD is regularized over. Computed based on simulated time series where retrievals with random noise inputs were compared with true simulated soil moisture and VOD time series. Computed given a 1.1K TB error standard deviation and average soil moisture of 0.2 m^3^ m^-3^.

Fig. S2. Same as Fig. 5, but including N = 3 regularization, where N equals the number of satellite overpasses that VOD is regularized over. Root mean square error of soil moisture and VOD for a simultaneous retrieval algorithm (DCA) and a VOD regularization algorithm (MT-DCA). Computed based on simulated time series where retrievals with random noise inputs were compared with true simulated soil moisture and VOD time series. Computed given a 1.1K TB error standard deviation and average soil moisture of 0.2 m^3^ m^-3^.

Fig. S3. Same as Fig. 8, but including N = 3 regularization, where N equals the number of satellite overpasses that VOD is regularized over. Correlation of high frequency variability between a simulated noisy time series and truth for soil moisture and VOD. Shown for both a simultaneous retrieval algorithm (DCA; N = 1 or no regularization) and a VOD regularization algorithm (MT-DCA; N>1 or with regularization). Computed based on simulated time series where retrievals with random noise inputs were compared with true simulated soil moisture and VOD time series. Computed given a 1.1K TB error standard deviation and average soil moisture of 0.2 m^3^ m^-3^.
